# Incidence of Neuroendocrine Tumors in the United States from 2001-2015: A United States Cancer Statistics Analysis of 50 States

**DOI:** 10.7759/cureus.4322

**Published:** 2019-03-26

**Authors:** Nicolas Patel, Bikramjit Benipal

**Affiliations:** 1 Internal Medicine, New York University School of Medicine, New York, USA; 2 Internal Medicine, Temple University, Philadelphia, USA

**Keywords:** gastroenterology, epidemiology, cancer, cancer epidemiology, neuroendocrine tumors, incidence

## Abstract

Introduction

Neuroendocrine tumors (NETs), once considered a rare malignancy, has started to become a more common cancer within the United States (US). Given the limited data available on the incidence of NETs in the entire US population, our goal in this study was to investigate the incidence of NETs in at-risk populations in all 50 states.

Methods

The United States Cancer Statistics (USCS) was used to obtain data for NETs from 2001 to 2015. An incidence analysis was done for sex, race, stage, primary location within the gastrointestinal (GI) tract, and US regional location.

Results

The overall incidence of NETs from 2001 to 2015 was 2.89 per 100,000 people per year. The overall incidence rates were the greatest for each stratification of males, blacks, localized disease, primary location in the small intestine, and in the Northeast. The incidence in males between 2013 and 2015 increased with an annual percent change (APC) of 8.44. Between 2006 and 2015, the incidence in blacks increased with an APC of 1.89. Between 2013 and 2015, the incidence of localized disease and a primary location in the small intestine increased with an APC of 16.89 and 14.37, respectively. In the Northeast, between 2013 and 2015, the incidence increased with an APC of 11.09.

Conclusion

In this study, we investigated the incidence of NETs using data obtained from the USCS database, which covers all 50 states. We found that there is a rising incidence in most subpopulations possibly related to improved compliance with surveillance colonoscopies and improved endoscopic and radiographic techniques. Further studies are needed to ultimately determine the exact causes of our findings. However, our study will serve as an important first step to determine the exact etiology for the rising incidence of NETs in all 50 states.

## Introduction

Neuroendocrine tumors (NETs), once considered a rare malignancy, have started to become a more prevalent cancer in the United States (US) [1]. The most common location of NETs is in the gastrointestinal (GI) system [1-3]. In the US, the incidence is increasing, with a seven-fold increase over the past 35 years [1]. Prior studies that have evaluated the incidence of NETs have used the National Cancer Institute’s (NCI) Surveillance, Epidemiology, and End Results (SEER) program. The SEER dataset represents nearly 28% of the US population [4]. Consequently, the SEER database can often misrepresent certain racial/ethnic groups and regions within the US [5]. The United States Cancer Statistics (USCS) database combines both the Center of Disease Control and Prevention’s (CDC) National Program of Cancer Registries (NPCR) and the SEER database to include data on all 50 states and thus can more accurately represent the US population [6]. In this study, we evaluated the incidence of NETs in all 50 states between 2001 and 2015, stratified by different risk factors.

## Materials and methods

Incidence data for NETs between 2001 and 2015 was obtained from the USCS database [7]. The USCS database provides the official federal statistics on cancer incidence and population data for all 50 states and the District of Columbia [5]. Cases were selected by primary site: C16.0 cardia, C16.1 fundus of stomach, C16.2 body of stomach, C16.3 gastric antrum, C16.4 pylorus, C16.5 lesser curvature of stomach not otherwise specified (NOS), C16.6 greater curvature of stomach NOS, C16.8 overlapping lesion of stomach, C16.9 stomach NOS, C17.0 duodenum, C17.1 jejunum, C17.2 ileum, C17.3 Meckel's diverticulum, C17.8 overlapping lesion of small intestine, C17.9 small intestine NOS, C18.0 cecum, C18.1 appendix, C18.2 ascending colon, C18.3 hepatic flexure of colon, C18.4 transverse colon, C18.5 splenic flexure of colon, C18.6 descending colon, C18.7 sigmoid colon, C18.8 overlapping lesion of colon, C18.9 colon NOS, C19.9 rectosigmoid junction, C20.9 rectum NOS, and C26.0 intestinal tract NOS. International Classification of Disease (ICD) for Oncology 3rd edition codes were used to extract data for carcinoid tumor (8240/3), enterochromaffin cell carcinoid (8241/3), adenocarcinoid tumor (8245/3), neuroendocrine carcinoma (8246/3), and atypical carcinoid tumor (8249/3). Incidence analysis was performed for sex, race, stage, primary site, and regional location within the US (Northeast, Midwest, South, and West). Race included whites, blacks, Asian or Pacific Islanders (API), and American Indian/Alaska Natives (AI/AN). The primary site included the stomach, the small intestine, and the colorectum. An incidence analysis was used by Tiwari et al., 2006, with modifications for confidence interval (CI) [8]. The Joinpoint Regression Program (version 4.5.0.1, DigitCompass LLC, Maryland, USA) was used to create incidence graphs and calculate annual percent change (APC) using the least square method [9]. Incidences were per 100,000 and were adjusted to the year 2000 US standard population. For all analyses, p<0.05 was considered statistically significant.

## Results

A total of 141,290 patients were included in the incidence analysis between 2001 and 2015 (Table 1). A total of 68,723 (48.6%) males and 72,567 (51.4%) females were included in the analysis. A total of 137,612 had an identifiable race at the time of diagnosis. Of those, 106,254 (77.2%) were white, 25,925 (18.8%) were black, 812 (0.60%) were AI/AN, and 4,621 (3.4%) were API. There was a total of 123,657 patients with an identifiable stage at the time of diagnosis. Of those, 72,246 (58.4%) were localized, 30,744 (24.9%) were regional, and 20,667 (16.7%) were distant. There was a total of 141,036 patients with a primary location of disease. Of those, 17,141 (12.2%) were in the stomach, 72,382 (51.3%) were in the small intestine, and 51,513 (36.5%) were in the colorectum. There was a total of 141,290 with an identifiable regional location within the US. Of those, 27,940 (19.8%) were in the Northeast, 32,678 (23.1%) were in the Midwest, 51,862 (36.7%) were in the South, and 28,810 (20.4%) were in the West.

**Table 1:** Patient Characteristics

Patient characteristics		
Gender (n = 141,290)		
	Count	Percent
Male	68,723	48.6%
Female	72,567	51.4%
Race (n = 137,612)		
	Count	Percent
White	106,254	77.2%
Black	25,925	18.8%
Asian or Pacific Islander	4,621	3.4%
American Indian/Alaska Native	812	0.6%
Stage (n = 123,657)		
	Count	Percent
Localized	72,246	58.4%
Regional	30,744	24.9%
Distant	20,667	16.7%
Primary Location (n = 141,036)		
	Count	Percent
Stomach	17,141	12.2%
Small Intestines	72,382	51.3%
Colorectum	51,513	36.5%
Regions (n = 141,290)		
	Count	Percent
Northeast	27,940	19.8%
Midwest	32,678	23.1%
South	51,862	36.7%
West	28,810	20.4%

**Figure 1:**
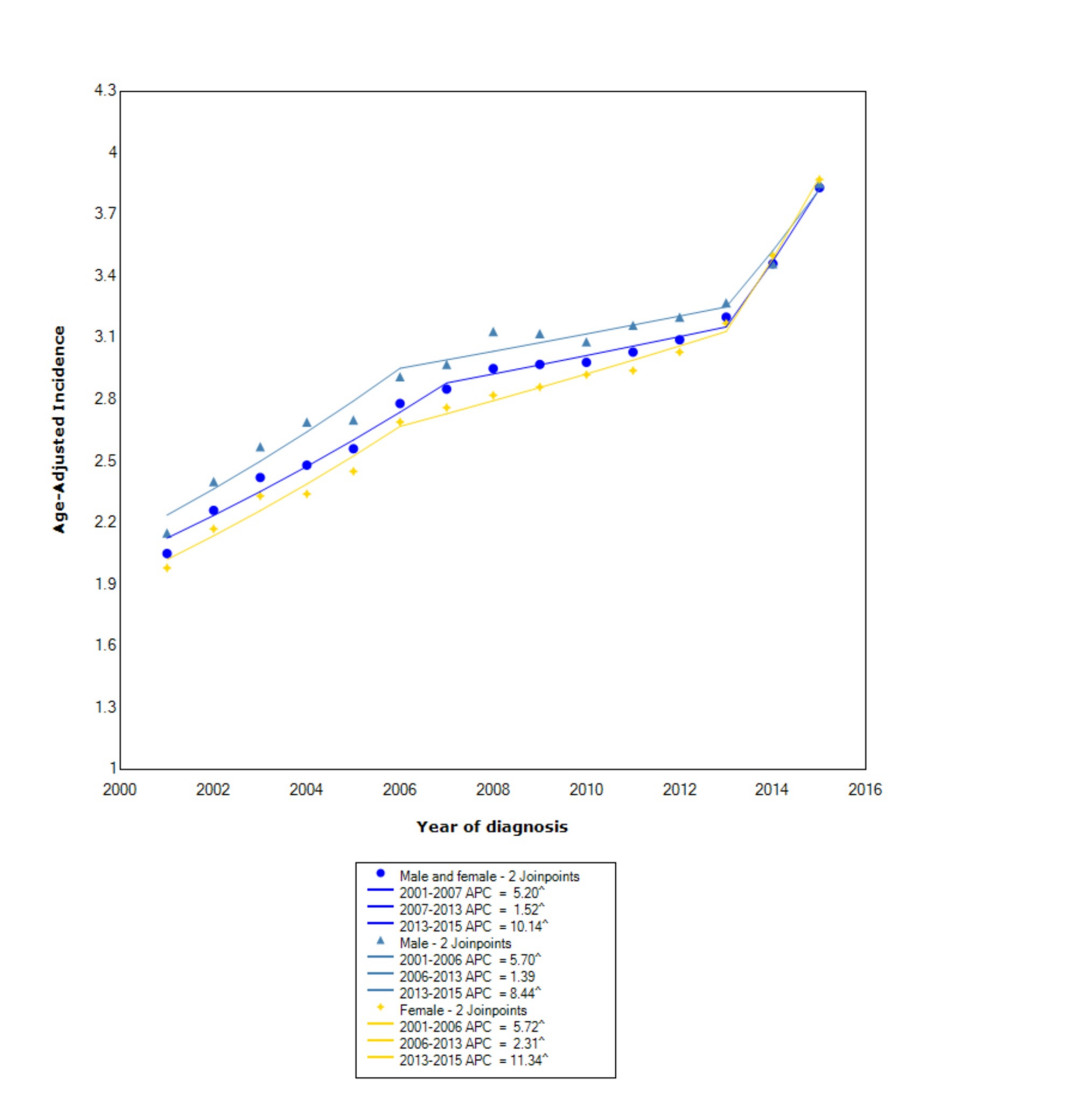
Incidence Rate, Sex ^ Indicates that the APC is significantly different from zero at the alpha = 0.05 Age-adjusted incidences are per 100,000 and age-adjusted to the 2000 United States standard population. APC: annual percent change

The overall incidence of NETs from 2001-2015 was 2.89 per 100,000 people per year. Males had an overall incidence rate of 3.01 (95% CI 2.98-3.03), which was greater than females, who had an incidence rate of 2.81 (95% CI 2.79-2.83). The incidence in males, between 2001 and 2006, increased with statistical significance (APC 5.70). Following that, between 2006 and 2013, the incidence increased at a slower rate (APC 1.39). However, between 2013 and 2015, the incidence started to increase at a significant rate with statistical significance (APC 8.44). Similarly, in females, between 2001 and 2006, the incidence was increasing with statistical significance (APC 5.72). Subsequently, between 2006 and 2013, the incidence continued to increase with statistical significance but no longer at the same rate (APC 2.31). Between 2013 and 2015, the incidence started to increase at a rapid rate with statistical significance (APC 11.34) (Figure 1).

**Figure 2:**
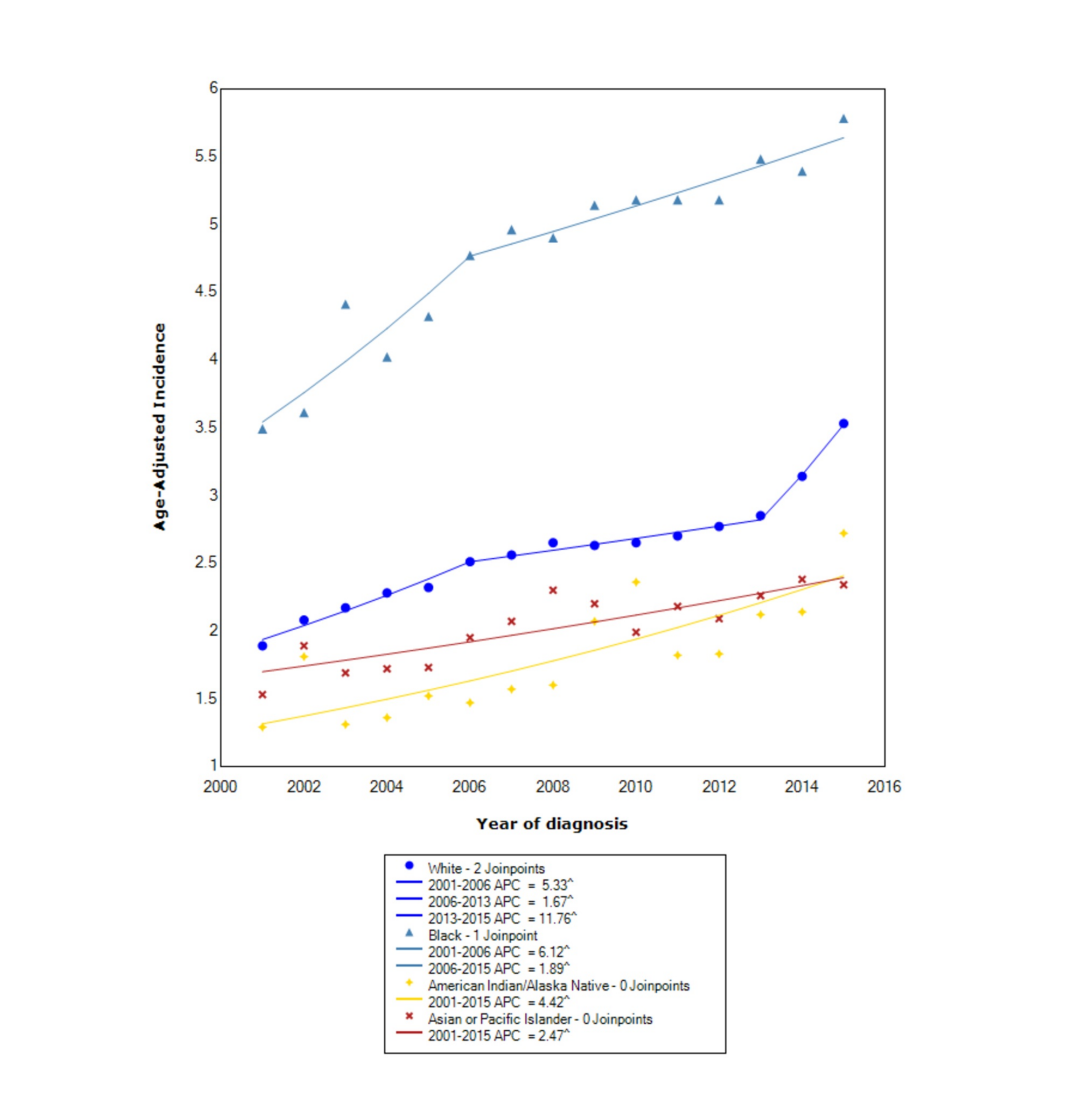
Incidence Rate, Race ^ Indicates that the APC is significantly different from zero at the alpha = 0.05 Age-adjusted incidences are per 100,000 and age-adjusted to the 2000 United States standard population. APC: annual percent change

When stratified by race, NETs had the greatest incidence in blacks (4.86 95% CI 4.80-4.92), followed by whites (2.60 95% CI 2.58-2.61), API (2.06 95% CI 2.00-2.12), and lastly AI/AN (1.85 95% CI 1.72-1.99). Between 2001 and 2006, the incidence in whites increased with statistical significance (APC 5.33). Subsequently, between 2006 and 2013, the incidence increased with statistical significance (APC 1.67); however, it was no longer at the same rate. After 2013, the incidence started to increase at a rapid rate with statistical significance (APC 11.76). In blacks, between 2001 and 2006, the incidence increased with statistical significance (APC 6.12); however, subsequently, after 2006, the incidence continued to increase with statistical significance but no longer at the same rate (APC 1.89). For AI/AN, between 2001 and 2015, the incidence increased with statistical significance (APC 4.42). For API, between 2001 and 2015, the incidence also increased with statistical significance (APC 2.47) (Figure 2).

**Figure 3:**
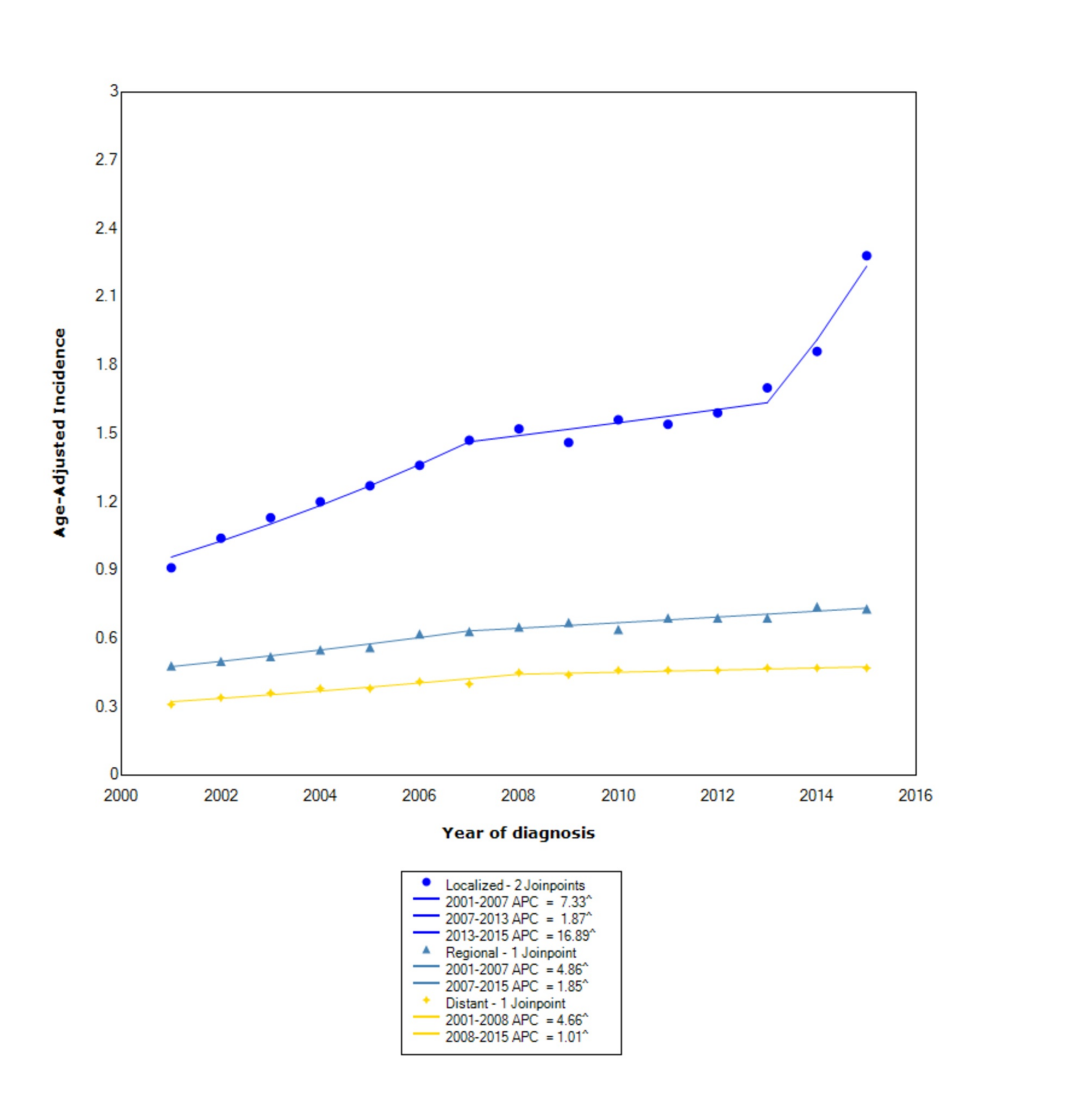
Incidence Rate, Stage ^ Indicates that the APC is significantly different from zero at the alpha = 0.05 Age-adjusted incidences are per 100,000 and age-adjusted to the 2000 United States standard population. APC: annual percent change

When comparing stage at diagnosis, NETs had the greatest incidence in those with localized disease (1.48 95% CI 1.46-1.49), followed by regional (0.63 95%CI 0.62-0.64), and, lastly, distant disease (0.42 95% CI 0.415-0.427). For localized disease, between 2001 and 2007, the incidence increased with statistical significance (APC 7.33). Between 2007 and 2013, the incidence continued to increase but no longer at the same rate (APC 1.87). Subsequently, after 2013, the incidence started to rise at a rapid rate (APC 16.89). For regional disease, between 2001 and 2007, the incidence increased with statistical significance (APC 4.86); however, after 2007, the incidence continued to increase but no longer at the same rate (APC 1.85). For distant disease, between 2001 and 2008, the incidence increased with statistical significance (APC 4.66); however, after 2008, the incidence continued to rise but no longer at the same rate (APC 1.01) (Figure 3).

**Figure 4:**
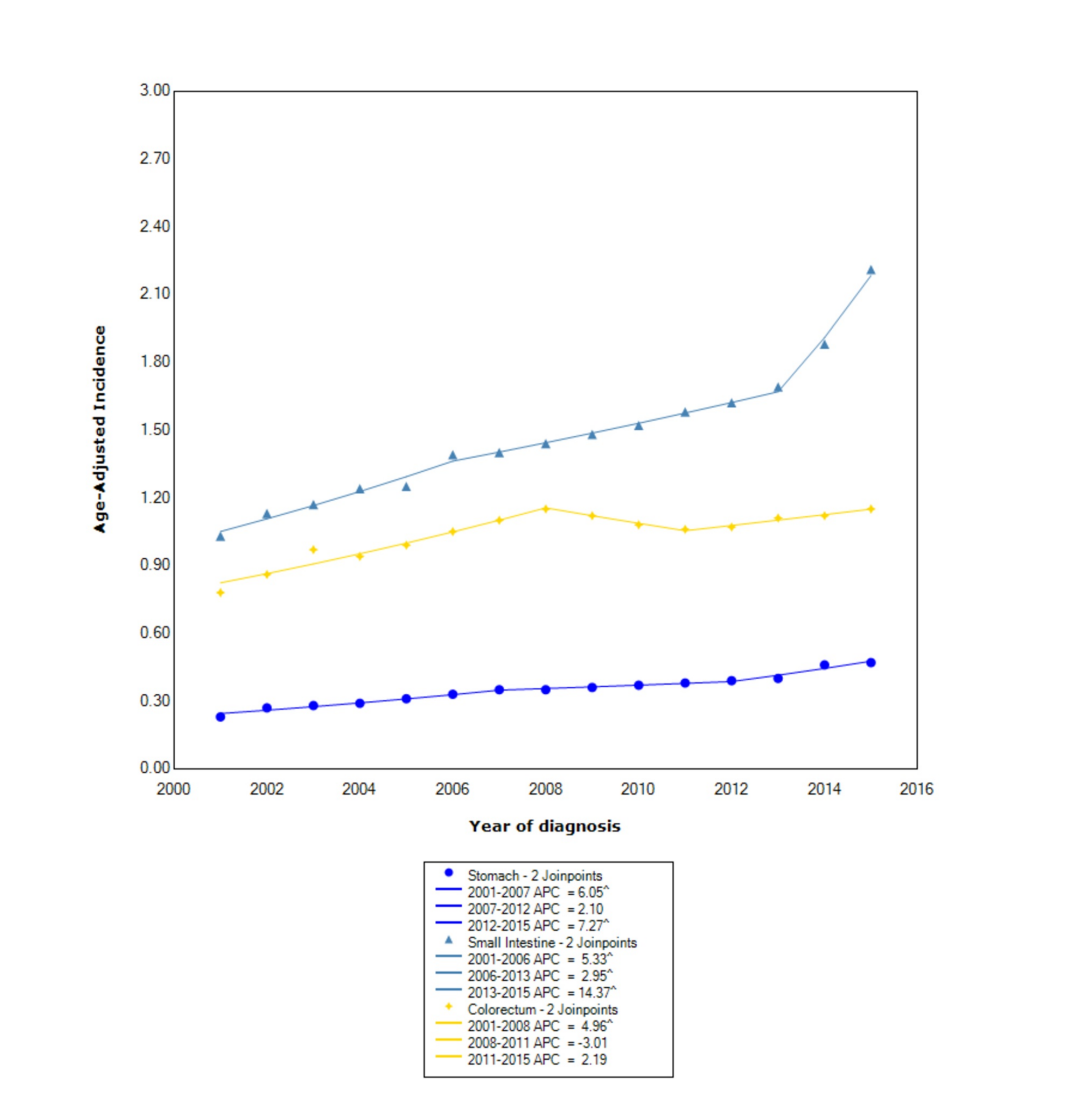
Incidence Rate, Primary Location ^ Indicates that the APC is significantly different from zero at the alpha = 0.05 Age-adjusted incidences are per 100,000 and age-adjusted to the 2000 United States standard population. APC: annual percent change

When stratifying incidence based on primary site of disease, the greatest incidence occurred in the small intestine (1.49 95% CI 1.47-1.50), followed by colorectum (1.04 95% CI 1.03-1.05), and, lastly, the stomach (0.354 95% CI 0.348-0.359). In the small intestine, between 2001 and 2006, the incidence was rising with statistical significance (APC 5.33); however, between 2006 and 2013, the incidence continued to rise but no longer at the same rate (APC 2.95). Between 2013 and 2015, the incidence started to rise at a considerably faster rate (APC 14.37). In the colorectum, between 2001 and 2008, the incidence was increasing (APC 4.96); however, between 2008 and 2011, the incidence started to decrease (APC -3.01). After 2011, the incidence started to increase again with an APC of 2.19. In the stomach, between 2001 and 2007, the incidence was rising with statistical significance (APC 6.05). Between 2007 and 2012, the incidence continued to increase but no longer at the same rate (APC 2.10). After 2012, the incidence started to increase again with an APC of 7.27 (Figure 4).

**Figure 5:**
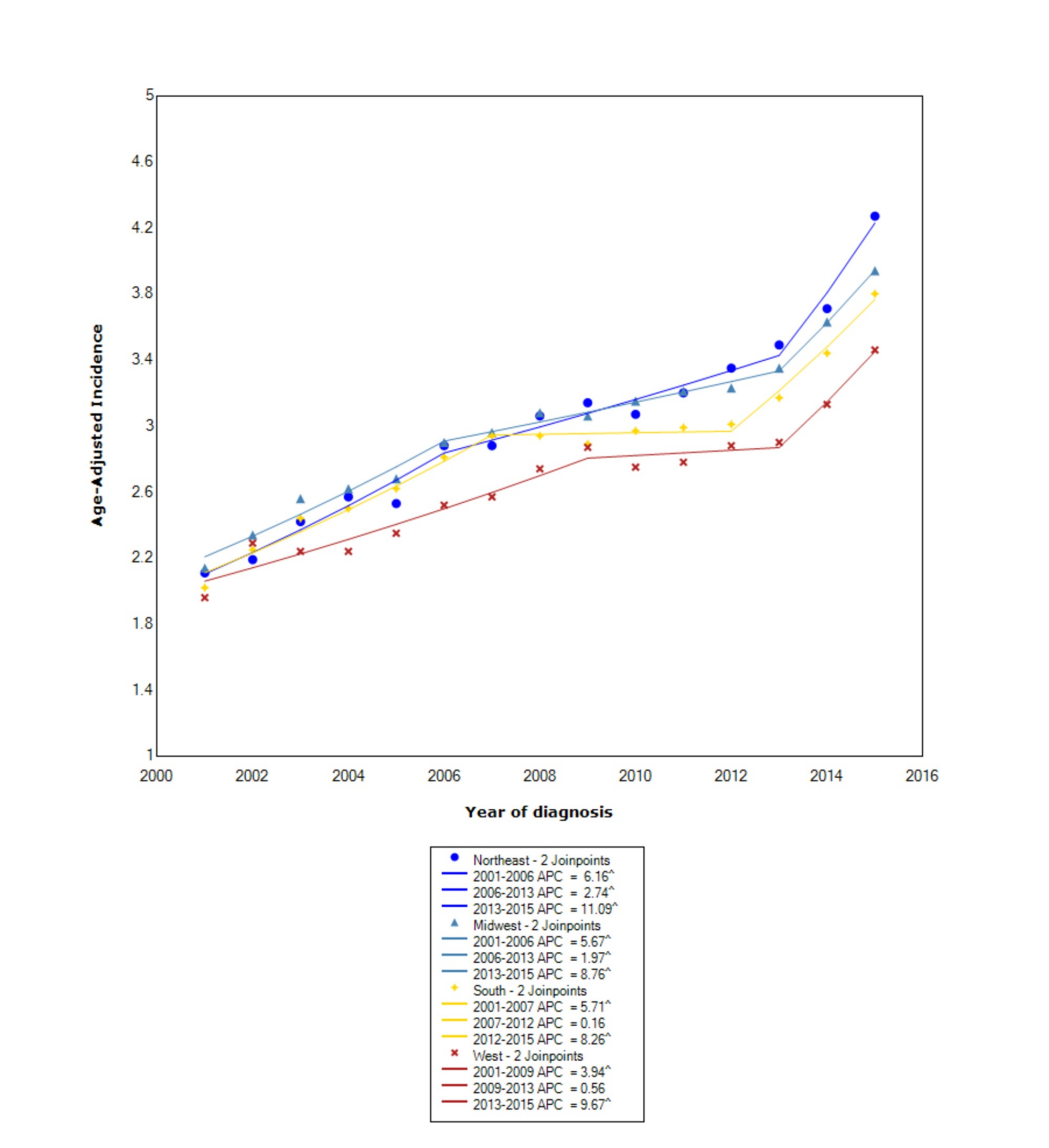
Incidence Rate, Region ^ Indicates that the APC is significantly different from zero at the alpha = 0.05 Age-adjusted incidences are per 100,000 and age-adjusted to the 2000 United States standard population. APC: annual percent change

When stratified by regional location within the US, the greatest incidence occurred in the Northeast (3.01 95% CI 2.97-3.04), followed by the Midwest (3.00 95% CI 2.97-3.04), the South (2.88 95% CI 2.86-2.91), and, lastly, the West (2.67 95% CI 2.64-2.70). In the Northeast, between 2001 and 2006, the incidence increased with statistical significance (APC 6.16); however, between 2006 and 2013, the incidence continued to increase but not at the same rate (APC 2.74). After 2013, the incidence started to increase at a rapid rate (APC 11.09). In the Midwest, between 2001 and 2006, the incidence was increasing with an APC of 5.67; however, between 2006 and 2013, the incidence continued to increase but no longer at the same rate (APC 1.97). After 2013, the incidence started to increase at a more rapid rate, with an APC of 8.76. In the South, between 2001 and 2007, the incidence was increasing with an APC of 5.71; however, between 2007 and 2012, the incidence leveled off with an APC of 0.16. After 2012, the incidence started to increase again at a rapid rate (APC 8.26). In the West, between 2001 and 2009, the incidence was increasing with an APC of 3.94; however, between 2009 and 2013, the incidence leveled off (APC 0.56). After 2013, the incidence started to increase at a rapid rate (APC 9.67) (Figure 5).

## Discussion

To the best of our knowledge, our study is the first to evaluate the incidence of NETs between 2001 and 2015 using the USCS database. We found an overall 1.07:1 male to female incidence ratio. Similar to prior studies, we also found that the incidence in males and females was increasing with similar trends [10]. However, unlike prior studies, we found that recently, after 2013, the incidence in females is increasing at a more rapid rate than in males. In the study by Tsikitis et al., when comparing race, they found that the incidence of rectal NETs was substantially greater in blacks than whites [10]. We found similar findings for overall NETs within the GI tract. Prior studies have attributed these findings possibly to the fact that minorities are more likely to have sigmoidoscopies performed at regular intervals in spite of the fact that whites undergo more frequent screening colonoscopies [10-11]. However, we also found that after 2013, the incidence of NETs began to increase at the greatest rate in whites and at the lowest rate in blacks, which might indicate a change in prior findings. In our study, we also found that the incidence of localized disease was the greatest overall as compared to other stages. However, we also found that after 2013, the incidence of localized disease was increasing at a more rapid rate than either regional or distant disease. Although the exact reasoning for these findings was not investigated, it may possibly be related to better compliance with screening colonoscopies and improved follow-up.

When examining the incidence of NETs based on primary location, prior studies have found that the incidence is increasing the most in the small intestine and colon [10,12]. We, however, found that the incidence was the greatest overall in the small intestine and colorectum; however, after approximately 2011, the incidence was increasing the greatest in the small intestine followed by the stomach. These findings may be the result of improved endoscopic and radiographic techniques; however, further research is needed to determine the exact cause [10]. When evaluating the incidence of NETs based on regional location within the US, we found that each region followed a similar trajectory. Moreover, all four regions had a rapid rise in incidence after approximately 2012. This data suggests that regional variations may only play a small part in the incidence of NETs. In other words, the variables causing the rising incidence are likely found throughout the US.

## Conclusions

In our study, we investigated the incidence of NETs in all 50 states. Our study showed that the overall incidence was greatest in males, blacks, localized disease, the small intestine, and in the Northeast. The APC in these groups increased rapidly between 2001 and 2015 and more so after approximately 2012. The reason for these findings is unclear and requires further studies; however, it appears that improved compliance with surveillance colonoscopies and advances in endoscopy and radiographic techniques may have some influence on our findings. Our study, to the best of our knowledge, is the first to examine the incidence of NETs in all 50 states using the USCS database. Our findings will serve as an important template for further studies to investigate the underlying causes of these findings.
